# A framework and practical approach to identify and address ethical issues arising in interventional health systems research

**DOI:** 10.1016/j.ijnurstu.2025.105263

**Published:** 2026-02

**Authors:** Edna Mutua, Dorothy Oluoch, Sebastian Fuller, Gloria Ngaiza, Nancy Odinga, Caroline Waithira, Vincent Kagonya, Onesmus Onyango, Naima Nasir, Gulraj Grewal, Abdulazeez Imam, Asma Rababeh, Michuki Maina, Kenneth Karumba, Maureen Kelley, Mike English, Sassy Molyneux

**Affiliations:** a KEMRI-Wellcome Trust Research Programme, Nairobi, Kenya; b Health Systems Collaborative, Nuffield Department of Medicine, University of Oxford, Oxford, UK; c Wake Forest University School of Medicine, Center for Bioethics, Health & Society, Winston-Salem, United States

**Keywords:** Embedded research, Neonatal care, Bystanders, Empirical ethics, Positionality

## Abstract

**Background:**

Embedded, interventional health systems research is increasingly promoted to better understand and strengthen the performance of health systems. However, for these forms of research, boundaries between clinical care, quality improvement, and public health can be blurred, and ethical implications and frameworks to draw upon are unclear. While there is evolving ethical guidance, few health systems studies have documented ethical dilemmas experienced post ethics approval, and the value of support processes introduced to manage arising dilemmas. In this discussion paper, we share our approach to handling the ethical dilemmas that arose while conducting embedded interventional health systems research in public-sector newborn units in Kenya.

**Methods:**

Building on our past research, and literature on debriefs, reflective learning, ethics reflection groups and moral case deliberations, we evolved an approach to holding regular structured ethics debriefs to discuss and agree upon how to handle ethical issues experienced during ‘fieldwork’. The research team maintained a ‘living log’ of all discussions, detailing all emerging ethical issues and any agreed actions. To prepare this paper, we conducted a thematic analysis of the living log and associated meeting minutes/recordings, and held a series of wider team meetings to reflect upon our learning.

**Findings and discussion:**

Numerous dilemmas were shared by research staff in our debrief fora. We grouped ethical issues encountered into 1) ‘bystander’ issues (defined here as background issues impacting health system functioning, facility staff, patients or families that were not caused or exacerbated by our research activities), 2) issues for those groups that were ‘research imposed’ and 3) issues related to the ‘comfort and well-being of research team members’. Most dilemmas raised related to feeling like bystanders in highly constrained health systems, complicated by our positionalities as ‘outsiders-within’, whereby as health researchers spending time in facilities we were neither fully ‘outsiders’ nor ‘insiders’ to the health system. There was constant moral labour involved in considering our responsibilities for action, which ranged from immediate action from a safety perspective, through rethinking how the research was conducted, to various forms of engagement and feedback across a web of stakeholders.

**Conclusion:**

The approach we developed offers a framework to assist research team members with the significant ethical dilemmas and challenges that arise over the course of conducting studies. We suggest activities to support working prospectively through emerging ethical dilemmas in future studies.

## What is already known

• Embedded, interventional health systems research has the potential to contribute valuable knowledge that supports system strengthening.
• Research ethics practice tends to focus on gaining study approval and adhering to protocols and regulations/guidance, leaving frontline staff to struggle alone in managing emerging ethical issues and tensions that arise during research.
• There are diverse practical and ethical challenges in gathering data for embedded health systems research, some of which are difficult to anticipate in advance.

## What this paper adds

• Insight into the ethical issues that arose over the course of conducting embedded, interventional health systems research in four newborn units in public sector hospitals in Kenya, and how we responded.
• An adapted framework and practical approach to gathering, categorising and responding to the ethical dilemmas that may be of value elsewhere.

## 1 Introduction

Health systems are complex social systems critical to the delivery of good quality, respectful care globally (Sheikh et al., 2014; Boga et al., 2025). Over the last two decades, Health Policy and Systems Research (referred to as ‘health systems research’ in this paper) has gained prominence as a field of research to better understand and strengthen the performance of health systems, with e*mbedded* forms of research increasingly emphasised (Ghaffar et al., 2017; Swaminathan et al., 2020). There are many different approaches to conducting embedded health systems research, but in all variants, researchers work inside or alongside a host organisation with the aim of strengthening learning about less visible, context-specific issues, and of promoting relevance and impact of research (Gilson et al., 2021). For these forms of research, the boundaries between clinical care, quality improvement, and public health can be blurred, and the ethical implications and frameworks to draw upon unclear (Luyckx et al., 2019).

Embedded health systems research may be particularly valuable in resource constrained settings, where health systems face both chronic, everyday stressors (resource constraints, constant policy change, staff turnover) and periodic sudden shocks (epidemics, climatic incidents, dramatic policy change and political upheaval) (Gilson et al., 2017). Given the complexity and context-specificity of many health system challenges, locally designed or at least locally tailored innovations and interventions are often essential. High quality embedded health systems research can be invaluable in informing these initiatives, but there are practical and ethical challenges throughout the research cycle, from study design, through science and ethics approval and study conduct, to ending the study. Ethics guidance is evolving to support health systems research across the research cycle (Faden et al., 2013; Pratt et al., 2017; Luyckx et al., 2019) with a central concern being establishing and maintaining appropriate relationships that support knowledge sharing across multiple stakeholders with potentially different interests and values (Molyneux et al., 2016; Jepkosgei et al., 2019).

Many ethical issues and dilemmas can be anticipated and built into study designs and plans from the outset. However, not all issues can be predicted in advance, and even where they are, the realities are not always fully appreciated until experienced (Farsides, 2019). Also, research ethics practice tends to focus on gaining study approval and adhering to protocols and regulations, leaving frontline staff to struggle alone in managing emerging ethical issues and tensions that arise in day-to-day research activities (Khirikoekkong et al., 2020, Nkosi et al., 2020, Steinert et al., 2021, Nzinga et al., 2023). The emotional toll can be high for those staff, and there can be negative implications for staff well-being, team relationships, stakeholder engagement and ultimately for the learning gained and the social value of the research (Nkosi et al., 2020; Molyneux et al., 2021; Nzinga et al., 2023; Boga et al., 2025). Researchers have called for better guidance and support in identifying and responding to ethical dilemmas that arise for frontline staff post ethical approval (Luyckx et al., 2017). Initiatives include participatory training in ethics practice (Kombe et al., 2019), collaborative development of staff safe-guarding policies and processes (Aktar et al., 2020), research distress protocols outlining procedures to follow where participants present with adverse reactions during interviews (Whitnet, 2022), and various forms of regular debriefing exercises which include a focused ethics component (Musschenga, 2005; Guillemin et al., 2009; Bruun et al., 2019b; Molyneux et al., 2021).

In previous work centred on a multisite, multidisciplinary clinical observational study, we developed an approach to help identify, unpack and respond to the ethical issues faced by frontline staff in their interactions with research participants, and especially vulnerable children and their family members (Molyneux et al., 2021). In this discussion paper we contribute to evaluating the usefulness of the approach for a very different study design: an embedded health systems interventional study which included adding staff into four public sector newborn units in Kenya. We share our approach to gathering ethical issues and concerns and our adapted framework to categorise issues based on our perceived responsibility to act and types of action. We illustrate the different types of issues faced and our responses, and in the discussion reflect upon the wider relevance of our work.

## 2 Study design and setting

### 2.1 Kenyan newborn units and the HIGH-Q study goals

**Fig. 1:**
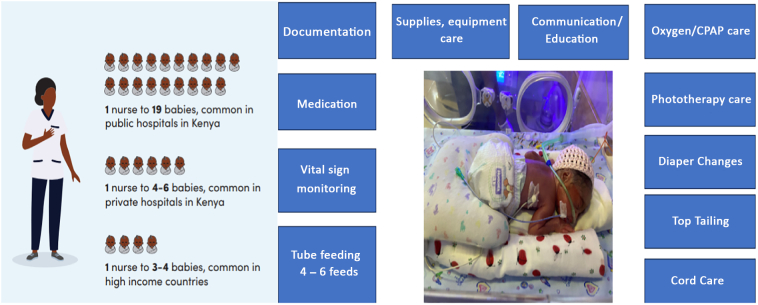
*Previous research across public, private and mission hospitals found that in public hospitals, nurses cared for a median of 19 babies per shift, with some responsible for more than 25 babies. Private hospitals fared slightly better (1:4–6), but these ratios still exceeded the recommended 1:3–4 ratio more commonly seen in HICs. The babies in nurses' care and their parents need nurses to perform numerous tasks.

Our work was conducted in Kenyan public hospital newborn units where prior work and engagement with stakeholders indicated that rigorous high quality intervention research was needed to support better decision-making in increasingly resource-constrained settings (Imam et al., 2022). Mortality rates are high, and staff, especially nurses, face huge day-to-day pressures linked to huge workloads (see Fig. 1), resource shortages, and environmental inadequacies (English et al., 2020; Mcknight et al., 2020). In these extremely charged settings, nurses are expected to provide medical interventions, ensure careful monitoring and infection prevention, initiate feeding and provide emotional support to parents. Unsurprisingly, high levels of stress and burnout, and significant communication challenges, have been documented (Musitia et al., 2022).

In collaboration with local and national stakeholders, we sought to evaluate a programmatic intervention to test additional workforce interventions and start to examine post-discharge care needs. The Learning to **H**arness **I**nnovation in **G**lobal **H**ealth for **Q**uality Care (HIGH – Q) Programme aimed to investigate how the introduction of new staff in Kenyan neonatal care units impacts care quality and the experiences of healthcare workers and families in the context of an existing program to provide neonatal technologies (Imam et al., 2022). The existing program began in 2019 through a partnership between NEST 360 (Newborn Essential Solutions and Technologies) and the Ministry of Health and 13 specific county hospitals. It included a package of technologies, associated training for health workers and local biomedical engineers, and promotion of local quality improvement through for example quarterly mentorship/support supervision visits (Maina et al., 2025).

### 2.2 HIGH-Q study design and methods

**Fig. 2:**
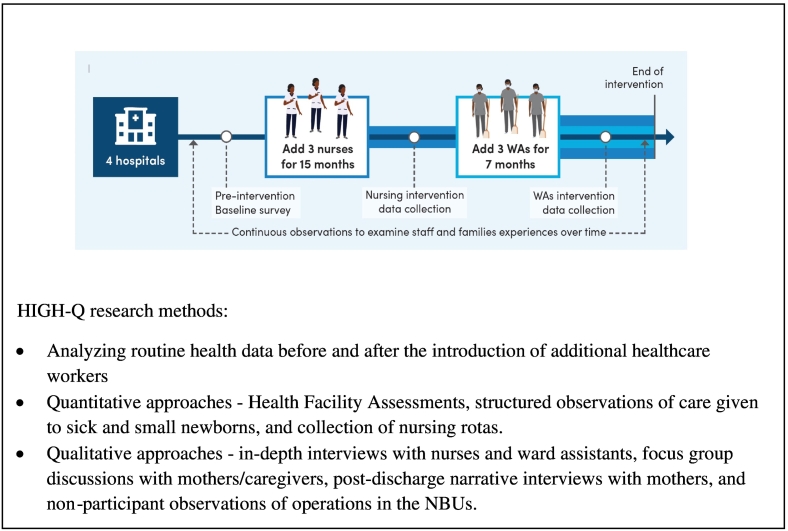
HIGH-Q study design and methods.

To achieve the HIGH-Q study aims, we co-designed a multidisciplinary study with inputs from national stakeholders including senior health policy makers, hospital managers and patient representative groups (Fig. 2) (Karumba et al., 2024; Maina et al., 2025).

A central element in HIGH-Q was a workforce intervention in which four of the 13 hospitals involved in the NEST360 program were selected as the recipients of an additional three nurses per hospital, expecting that by 15 months this extra staffing would have a measurable effect on nursing care delivered. Given that many nurses may also carry out many non-technical tasks as part of their duties, three ward assistants were then introduced into the same units 8 months after the three nurses, for 7 months, to free up nurses' time enabling them to carry out key nursing tasks which are often missed (Maina et al., 2025).

We were cognisant in designing our study that there might be unintended negative consequences of introducing new staff funded as part of a research programme into some facilities. We therefore carefully discussed with national, county and facility managers how staff would be employed, paid and managed with each hospital in advance (Karumba et al., 2024). Throughout the research we continuously checked with stakeholders that we were not unduly burdening our participants (individuals and facilities). In these ways we were seeking to adhere to ethical concerns of maximising learning and benefits while minimising disruption, harm and waste.

The HIGH-Q primary data collection team were primarily from Kenya and other African countries. There were 2 paediatricians, 3 nurses, 2 social scientists and 2 global health specialists in the team, supported by temporary nutritionists selected in collaboration with sampled public health facilities.

### 2.3 Regular team debriefs including reflecting on ethical dilemmas

**Fig. 3:**
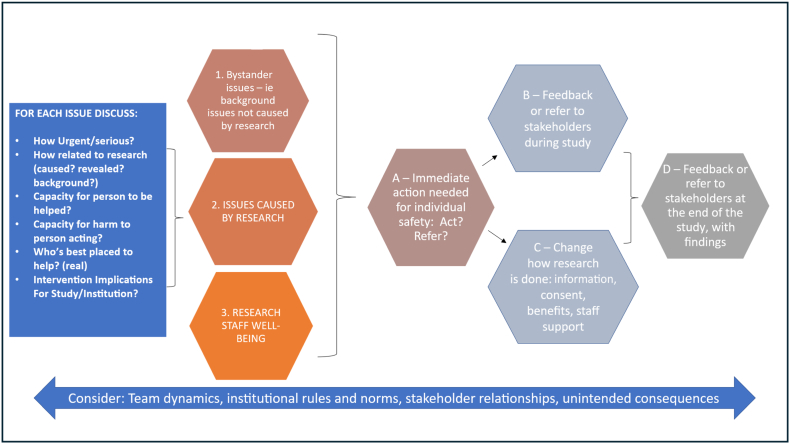
Our approach to gathering, categorising and responding to ethical issues.

We were cognisant in designing our HIGH-Q study that during data collection, especially observations, researchers might experience situations of ethical concern. We had plans in place on how to handle expected issues, and introduced monthly team debriefs, each of one to two hours, to specifically discuss any emerging ethical dilemmas or concerns. We defined ethical dilemmas as situations where team members were not sure what the right thing to do was, where they felt they knew what should be done but could not do it, or simply felt emotionally uncomfortable. Within meetings, we sought to set up an environment to allow supportive critical reflection on the issues experienced. To structure the conversations, we adapted the group-based ethics reflection approach developed in the earlier previous work (Molyneux et al., 2021); an approach in which we had drawn on the benefit-sharing and ancillary care literature and particularly Richardson and Belsky's partial entrustment model (Richardson and Belsky, 2004). Over several meetings, we tested the value of the approach and evolved it to the version summarised in Fig. 3 below.

As in our previous work (Nzinga et al., 2023), for each issue raised, a description of the issue was first provided by the person who experienced it, and then as a team we sought to analyse the situation, understand the issue and consider any research team responsibilities for action. We asked ourselves questions such as how urgent and serious is the issue, how much is it caused by or related to the research, what is the capacity of the person or facility to be helped, and are members of the research team the best people to intervene? (Fig. 3, left, blue). We weighed these considerations against potential unintended risks and consequences in the short and long term, including to babies, parents, staff, relationships and the research. We sought to ensure that the meetings were a safe space to allow team members with different backgrounds and experience to raise concerns; maximising learning from one another.

We broadly grouped ethical issues encountered into three categories (Fig. 3, centre left): 1) those related to the background situation that the research was being conducted in (in this case terming these ‘bystander issues (Sabin et al., 2019); which we defined as where research staff witness challenging health system functioning and staff and family realities, without having contributed to them through research activities or having a formal caring responsibility); 2) those caused or exacerbated by the research activities (‘research imposed’); and 3) those related to the comfort and well-being of the research team (‘research staff well-being’). Regarding responsibility for action (Fig. 3, right), we first identified whether these were: a) potentially critical or urgent, where the researcher felt something must be done immediately from a safety perspective; and/or b) issues that were not urgent but needed action by the team before completion of the research – for example ensuring they were included in periodic feedback meetings with the ward/hospitals, or to stakeholders with the power to act (such as the NEST360 team); or c) changing the way research is done – such as where and how consent is administered, how the team functions or what kinds of support are given to team members. Finally, was d) issues where we felt there was little that we could do beyond highlighting them and advocating for change in future publications, policy briefs, and meetings. Even where issues had been dealt with (Fig. 3, boxes a to c) these might feed into overall learning (box d). Regarding feedback, we carefully considered the timing, content and mode of delivery to minimise potential unintended consequences.

EM maintained a ‘living log’ constituting all reported emerging ethical issues and any agreed actions. To prepare this discussion paper, EM, DO and NO conducted a thematic analysis of the living log and associated meeting minutes/recordings (Braun and Clarke, 2012), and then organised wider team meetings (also including the remaining authors) to discuss each theme. In so doing we considered Smith’s four domains of critical reflection (Smith, 2011) as they applied in our context: the personal domain in relation to individual researchers thoughts and actions in response to emerging operational and ethical challenges; the interpersonal domain in relation to our interdisciplinarity and interpersonal relations between the researchers and with other stakeholders; the contextual domain to focus on our knowledge of how newborn units operate and their interactions with the project as guided by our scope of work, inclusive of the data collection methods employed; and the critical domain to focus on the political, ethical, and social context that the project was operating under, cognisant of the power relations between researchers and other stakeholders and their implications on the people involved directly or indirectly.

## 3 Ethics statement

Ethical approval for the HIGH-Q study was obtained from the Scientific Ethics Review Unit (SERU) of KEMRI (Ref: KEMRI/SERU/CGMR-C/229/4203), and the University of Oxford, UK (Oxford Tropical Research Ethics Committee- Approval reference 26-21) and from the County Departments of Health of the four participating intervention hospitals.

## 4 Findings

We held a total of twelve dedicated ethics reflection debriefs across two main data collection periods, each of one to two hours, primarily in person but with some participants contributing through Microsoft Teams®. We illustrate some of the main sets of ethical issues linked to three main categories on the left of Fig. 3 in turn below, before then going on to describe how we responded (Fig. 3, right).

### 4.1 Bystander issues

There were many issues that the research staff raised in ethics debrief sessions that they found difficult to observe but that were essential background issues not caused by the HIGH-Q activities or staff. Research staff wondered what they could and should do to intervene. We share illustrative examples of issues in relation to communication and interactions between staff and parents, care processes and technologies, and data recording.

Regarding *communication and interactions between staff and parents* researchers observed disrespectful communication, where some healthcare workers shouted, scolded, or talked rudely to mothers in ways which appeared to offend them and negatively affect staff-parent relationships. There was inadequate information, with mothers often requesting researchers to provide information or assistance in contacting health workers, and researchers also observed some sensitive or private medical information being discussed publicly, such as a mothers HIV status or the loss of a baby. These situations were disrespectful and could be upsetting or humiliating for mothers. Mothers privacy and dignity was also undermined by space restrictions and inadequate quality gowns, with the latter sometimes ripped and revealing. All of these factors we felt undermined mothers emotional well-being and the provision of respectful care.

Our researchers described discomfort observing *care processes (see for example* Box 1). Mothers were only allowed to be present in the ward during three hourly feeding times and outside of these times, babies cries were often unattended, which stressed mothers. Where mothers were present, they witnessed frightening symptoms and procedures (convulsions, resuscitations, repeated efforts to draw blood and insert lines), with little or no emotional or practical support. Given staffing constraints, tasks were routinely delegated by busy nurses to students, with limited communication with families on what was happening and why. Sub-optimal technical quality of care was sometimes observed by research staff and mothers, such as misplaced nasal prongs or oxygen masks. Parents or staff sometimes asked research staff to intervene beyond their agreed roles in wards (for example to hold a baby or assist with clinical care), introducing dilemmas on if and how to respond. For staff with a clinical background, intervention dilemmas were particularly acute, especially when a baby was clearly deteriorating or a student was struggling with a procedure or treatment plan.

Box 1 **Babies removing oxygen masks and eye covers.**

**Table:** 

Today, three mothers told me they feel nurses are responsible for monitoring the children. They all said they feel bad when they find their babies' oxygen masks in the wrong place. They wonder if the babies benefit from the oxygen since most of the time the masks are off. They also feel bad when they see the eye cover for the babies using phototherapy out of place.

Alt-text: Box 1

*Machines* were in some cases observed to be malfunctioning and some *supplies* were out of stock. Nurses reported this to our research staff with the hope or expectation that we would replace or fix them. A final particularly challenging observation was incidences of inaccurate reporting of *clinical data*, whereby things recorded as having been done, for instance, pulse oximetry readings and temperature checks, and had not been done.

### 4.2 Issues caused or exacerbated by research

Research staff raised issues in ethics debrief sessions that, unlike the previous section, appeared to be either caused or exacerbated by our research activities (observations, interviews, or addition of new staff).

#### 4.2.1 Observations and interviews

We were aware from the outset of the *potential disruption to routine ward processes* by our presence, and the importance of organising the research teams in ways to minimise this. We were also worried that there would be concerns from staff that we were there to audit or judge them. We therefore intentionally factored in a first phase of immersing our research team in the context, to begin to understand routines, build connections, and interact with staff and caregivers. Formal data collection began only once the different players appeared comfortable with our presence. Although this approach appeared to work well, our ethics debriefs revealed we had to make changes to our initial research plans to ensure we did not contribute to ward overcrowding and disrupt service delivery.

*Practical challenges in consenting processes* were regularly shared in the ethics debriefs, despite their careful design at proposal development stages. Consent processes included verbal consent for unstructured observations and written consent for interviews and structured quantitative observations. For interviews conducted outside work hours (for staff) and in homes (mothers), there was compensation for time, in line with institutional guidance. Examples of challenges raised in debriefs included mothers finding the observation work hard to understand, and especially why consent was sought in writing for the more detailed and systematic quantitative observations. Also, despite explanations of research goals, research staff were still often being asked for help, as noted above. A further consent related concern raised in debriefs was that the compensation for interviews may have been an incentive for mothers and staff to participate, with some encouraging others to participate in order to get the compensation.

A specific challenge linked to quantitative observers was *heightened expectations and anxiety* among mothers. Here, observers spent long hours observing specific babies daily. Mothers occasionally interpreted this close observation as “looking out for the baby” with an apparent assumption that their babies would receive the required treatment and care and that the mothers could therefore “leave” the babies in the care of the observers. Several observers reported that mothers had approached them wanting the observers explain the condition of their babies to family members. In a contrasting ethical challenge, three critically ill babies whose parents had consented to quantitative observation unfortunately passed away in quite short succession in one facility. This was not only very difficult for the observers (see below) but also contributed to rumours in the ward that these deaths may have been linked to the observation in some way; mothers began to fear the research.

#### 4.2.2 The intervention arm of the study

In ethics debriefs, research staff raised and discussed several intervention-related challenges. With the introduction of additional nurses, we observed tensions between some new and resident nurses in one facility, linked to the new nurses taking on a nursing task that was generally left to paediatricians (although these tensions eased over time as the new nurses gained respect and built relationships with resident nurses). In one hospital, the addition of ward assistants to take on non-clinical nursing tasks, aimed at freeing up nursing time to enable them to offer better care, also introduced tensions between ward assistants and nurses, and between past and new ward assistants. An intervention related concern of the research team from the outset, and periodically through debriefs, was the effect of the withdrawal of additional nurses and ward assistants at the end of the intervention period.

### 4.3 The comfort and well-being of the research team

As suggested through the previous sections, our research team members experienced discomfort and some significant distress, related to observing very difficult situations for babies, family members and staff (see for example Box 2), and being unable, often, to respond to requests for help with information, care or resources.

Box 2 **The researcher left alone in the ward.**

**Table:** 

The afternoon shift had one nurse on duty. There was no student nurse. The nurse did not attend to any of the babies because she had a lot of work to do. She did not take the vitals either. She also found herself going to the billing office to process files for discharge. She went twice, and at some point, I was left in the ward alone because mothers were on her case and wanted their files so that they could be discharged to go home. They kept coming to check whether the files had been returned from the billing office. I asked [the nurse] what she would have done if she had a critical baby; she told me she would have forgotten about the discharges and attended to the baby.

Alt-text: Box 2

Particularly difficult situations to witness were the deterioration and deaths of babies, and families receiving very little support with their anxiety and grief. System pressures sometimes meant the bodies of deceased babies remained on the ward while awaiting formal transfer to the mortuary. This could result in additional distress for mothers, staff and researchers. Our research staff felt unable to act, beyond comforting mothers where appropriate. Their concerns were heightened when rumours were circulating that structured observations had contributed to the babies' deaths, requiring immediate action by the study team, as discussed below.

Home based interviews also presented challenges, including mothers explicitly asking for help with health or financial matters. There were concerns among our team in considering their responses about setting precedents, introducing inequities between families and going against institutional guidance. We also came across families offering for instance to prepare special meals, raising concerns about either adding to family vulnerabilities (through accepting) or causing offence (through refusing).

## 5 How we responded to ethical issues

For all of these issues we *drew on our evolving framework to consider researchers responsibilities*. In all cases we were mindful of busy nurses working in a very challenging environment, and of power-hierarchies within wards and between staff and parents, all of which we did not want to exacerbate. We did not want staff to feel blamed, judged or undermined, to add to their already heavy workloads with interruptions and requests, or to undermine broader relations by introducing concerns that we were judging or testing them or intervening as family representatives. We were also aware that those of us who are non-clinician researchers are not always best placed to identify critical from non-critical situations, and even when we are clinically trained, we were not employed in those settings to provide care. On the other hand, we also did not want clear safety issues with an obvious and actionable intervention to be ignored, to be inhumane in how we interacted with mothers or students facing extreme distress, or to be unhelpful to over-stretched staff in moments we could assist.

### 5.1 Immediate action needed for safety (Fig. 3, box A)

There were some *bystander issues* – primarily safety related - where we agreed we should immediately intervene. For example, where the oxygen mask and eye cover during phototherapy was not in place or where a baby's hands or leg was in harms way, we agreed that researchers could discretely adjust it or gently ask a student to correct the problem. For more technical issues and situations requiring skilled staff to intervene fast, we encouraged researchers to immediately notify a nurse on duty, for instance if a baby was vomiting.

In relation to the *issues caused by research*, all of these required some form of action by the team. However, only those related to the specific challenges linked to quantitative observers of *heightened expectations and anxiety* among mothers were considered to require immediate urgent action. Expectations that non-trained observers were “looking out for the baby” and rumours that observations were causing deaths required urgent clarification of roles to minimise expectations and risks of unintended negative outcomes. Beyond individual explanations, as a team we organised further engagement meetings in the facility where rumours were circulating to re-build relationships and trust with mothers. Concerning the exit of additional nurses and ward assistants at the end of the study period, research team members advocated for their retention in facilities during meetings with decision-makers, but acknowledged they had no control over final decisions.

### 5.2 No action needed immediately – Fig. 3, box B and D

For more routine *bystander issues* without an immediate safety implication we agreed that these should largely be documented as part of understanding the research context, and in some cases followed up as part of feedback processes to wards or to others stakeholders. Regarding how the wards are organised, and most communication issues, we felt we could share our learning and recommendations as part of the research outcomes (Fig. 3, right D). Messages could be produced collaboratively with colleagues and across facilities in ways that did not inappropriately point fingers to support greater positive impact in the short and longer terms. Examples of issues where earlier feedback was agreed (Fig. 3, box B) include informing NEST 360 about equipment they were responsible for that needed repair (whether observed by them or raised by clinical staff), and highlighting general questions and concerns mothers had that staff may not have been aware of.

Where hospitals asked us for equipment, we generally reiterated that we were unable to provide items. In managing these requests and expectations we sought to be honest about our roles and the limitations of what we could provide, focused on seeking to understand realities for staff and parents and track the impacts of the additional staff provided. We were balancing trying to avoid being seen as resource providers for a highly constrained system with maintaining research relationships and learning in the medium and longer term.

### 5.3 Improve the way the research is done – Fig. 3, box C

For all *issues caused by research*, some form of action was needed. To minimise disruption to ward routines, we designed a new schedule to alternate the qualitative and quantitative observation teams presence, and agreed with health workers to interview them in a small office/sitting space within the newborn unit. Regarding consent, the importance of a continuous process of gently re-explaining our work wherever possible was underscored. We discussed making changes to the compensation approach but did not consider the funds to be an undue influence, or to be inappropriately disruptive of relationships or data quality. Regarding relationships between nurses and new ward assistants, we learned that these arose from county/hospital level recruitment and employment structures and processes, which were beyond the control of the research project (but important to document as part of study learning).

In ethics debriefs, and in related studies, we realised that some of the *bystander issues* and associated discomfort and *distress among research staff* were linked to nurses receiving inadequate training and support in communication and management of emotions, including grief management. We were able to intervene as a research team through feeding into an on-going health worker communication and emotional support intervention in the hope that the training would also assist clinical staff and parents in future (Boga et al., 2025). *Research staff* were supported through the debriefs and individually with line managers to work through ethical challenges, which appeared to provide some relief.

## 6 Discussion

We developed a framework and approach to assist research team members with discussion and decision-making on the practical and emotional challenges that arose over the course of conducting an embedded, interventional health systems study.

Most dilemmas we faced related to feeling like ‘bystanders’ in interactions among staff and between staff and parents in highly constrained health systems. We use the term ‘bystanders’, drawing in part on Sabin et al.s paper (Sabin et al., 2019), as situations where research staff witnessed challenging health system functioning and staff and family realities, without having contributed to them through research activities or having a formal caring responsibility. For these issues staff sometimes felt a deep moral responsibility or felt need to respond or act, but were anxious about if and how to do so given their limited roles and training and the potential to cause unintended physical or social harm. We are aware in our use of the term bystanders that there are grey areas and overlaps with ancillary care and associated obligations (Richardson and Belsky, 2004; Hyder and Merritt, 2009). In many of the ‘bystander scenarios we report, we judged our ability to intervene in the short term without introducing unintended harms or disadvantages to patients, families, staff or the research goals as relatively minimal. Immediate actions included minor interventions, referrals to clinicians to address safety concerns, and feeding into staff training programmes to support change in the short and longer term (Boga et al., 2025). Medium term actions centred on careful group level feedback in regular facility level meetings to build awareness and hopefully positive context relevant responses.

Other ethical issues arising were either caused by or exacerbated by our various research activities, placing a far greater responsibility on us to act immediately in for example, changing or re-explaining research activities. For research staff, there was a large emotional toll in conducting the work and the constant moral and practical work of navigating complex issues and relationships. Some relief was provided through our debrief approach and follow-ups and through individual support from immediate supervisors.

Many of the types of ethical issues we encountered have been observed elsewhere (Hyder and Merritt, 2009; Dixon-Woods and Bosk, 2010; Jepkosgei et al., 2019; Sabin et al., 2019), and had been discussed in advance, with broad agreements in place on how to handle them. However, as noted by Farsides, it is ‘only when you get inside [that] you really discover what is troubling a workforce at a particular time, what people care most about…’ (Farsides, 2019). Also whether or not we were really bystanders at all, and associated decisions on if, when and how to act, were complicated by our complex positionalities as ‘outsiders-within’ (Dixon-Woods and Bosk, 2010). Conducting a large multi-disciplinary *embedded health systems* study, with a human resource *intervention arm,* we were neither fully ‘outsiders’ nor ‘insiders’, and our positionalities across the research team varied, linked to training, experience and roles (clinicians/social scientists, familiar or not with newborn units, quantitative/qualitative observer or interviewer, hospital/community based, access to health managers). Our initial approach to handling anticipated and emerging ethical issues had to be adapted and refined. In practice, as described by Dixon-Woods and Bosk, we were seeking to maximise learning and social value through balancing: 1) establishing and maintaining relationships that support access and knowledge sharing (gaining insider status); while 2) adhering to study protocols, avoiding coming to see questionable practices as normal or acceptable, and avoiding feeling uncomfortable about ‘betraying’ staff who gave us access (maintaining outsider status) (Dixon-Woods and Bosk, 2010). While these types of labour are crucial in large research programmes, as well as in global health more generally, they are often rendered invisible (Nkosi et al., 2020; Zakayo et al., 2020; Kingori et al., 2022; Khirikoekkong et al., 2023).

As observed for similar approaches elsewhere (Bruun et al., 2019a, Bruun et al., 2019b; Molyneux et al., 2021), our debrief sessions and associated responses did not always result in unequivocal solutions. However, in sharing the moral labour of frontline staff and their immediate managers we add visibility to this complex work and hope to contribute to wider consideration of approaches with potential to minimise the significant moral distress associated with keeping dilemmas and anxieties private or pushing them aside. Our experiences support the value of setting up regular ethics discussions as a core component of ethical practice in interventional health systems research, alongside aligning the design with emerging ethics guidance for embedded implementation research. The latter includes comprehensive stakeholder engagement, transparency on sustainability of successful intervention elements, careful consideration about from whom and how informed consent should be obtained, and balancing of disadvantages and benefits to different groups (Molyneux et al., 2016; Pratt et al., 2017; Jepkosgei et al., 2019; Luyckx et al., 2019). These processes were followed for HIGH-Q, informed by institutional and national guidance on consent, engagement and benefit-sharing, as outlined in detail elsewhere (Musschenga, 2005; Guillemin et al., 2009). Our framework and approach required a good understanding of local processes and resources (to avoid referrals ‘to nowhere’ (Khirikoekkong et al., 2020)), and strong collaboration and prior engagement with local stakeholders (to ensure action pathways were clear and agreed). We also reassured staff that they have to respond to a situation as they felt best at a given time, given that not all scenarios can be predicted in advance and each researcher is different.

### 6.1 Implications for research

Drawing on the literature and our experiences, we have a number of recommendations to support working prospectively through emerging ethical dilemmas in future similar studies, all of which require time, resources and careful attention to socio-cultural and organisational norms, hierarchies, structures and rules. These recommendations assume the overall study design has been carefully designed to align with emerging ethics guidance for embedded implementation research, and related approaches such as learning health systems (Faden et al., 2013) and ethnography (Creswell and Creswell, 2017).

As part of *fieldwork planning and training processes*, consider with research team members and others with deep insight into the research context, the ethical and emotional issues and dilemmas that might arise in the field, and discuss and agree provisional approaches to handling them. Fig. 3 may assist with categorisation of potential issues, and responses. Depending on the specific study design, topic and context, it may be possible to develop detailed resource lists and referral agreements, and to text out very specific responses by research staff to expected scenarios (as in some distress protocols such as Whitnet, 2022). The ability to intervene as bystanders will likely be influenced by positionality of team members (particularly whether they are clinical or not; nationals or other nationalities), the specific issue (for example whether it involves any direct interaction with a patient/parent), and by institutional and national laws and ethical guidance.

*During the fieldwork*, incorporate emotional/ethical issues into debrief sessions or establish sessions specifically devoted to these dimensions of the work, to revisit and revise prior agreements as needed. This requires establishing safe spaces where team members feel free to raise and discuss issues, which is far from straightforward in busy research programmes with pressure to achieve targets to meet funding agreements. Careful facilitation and strong engagement and support from senior study staff are essential. Agreed processes need to align with and inform study consent, engagement, ancillary care and benefit-sharing plans, and institutional guidance, and may require amendments to research proposals, and in turn ethics approval.

Any formal, group-based activities will have to be supplemented by individuals in the field being able to raise issues one on one with immediate supervisors, and have support with urgent decision-making in emergency situations. The latter points to the challenging roles that immediate supervisors in research hierarchies play in offering practical and emotional support across large teams and filtering what information is shared with more senior staff (Nzinga et al., 2021). They are likely to need preparation and support in what are essentially complex leadership roles with critical implications for stakeholder engagement, frontline staff well-being, ethical practice and data quality.

### 6.2 Strengths and limitations

This paper presents an account of the ethical dilemmas we encountered, and the practical steps taken, drawing on information recorded as part of the process. Future research could include prospectively designed studies to examine the issues and evaluate the debrief approach more formally, including differences in opinions and experiences across stakeholders. Some of the issues raised in our sessions, and responses will be specific to the study design and team, methodological approach, and geographical and organisational context. However, the literature and our past experiences suggest many health research teams operating within resource constrained health systems will be juggling research and healthcare responsibilities and struggling to cope in structurally and emotionally challenging contexts.

## 7 Conclusion

We built upon an existing framework and approach to assist research team members with the practical and emotional challenges that arise over the course of conducting embedded, interventional health systems research. Most dilemmas we faced related to being ‘bystanders’ in interactions among staff and between staff and parents in highly constrained health systems. Further ethical issues arising were either caused or exacerbated by our research activities, placing an emotional toll on our frontline research staff and their immediate managers. Our debrief sessions interfaced with our study design, stakeholder engagement and consent processes, benefit sharing and referral agreements, and staff training to support ethical practice and quality of learning. We would value feedback from others on the potential relevance of adapting our framework and approach elsewhere.

## CRediT authorship contribution statement

**Edna Mutua:** Writing – review & editing, Writing – original draft, Supervision, Methodology, Investigation, Formal analysis, Data curation, Conceptualization. **Dorothy Oluoch:** Writing – review & editing, Supervision, Methodology, Investigation, Formal analysis, Data curation, Conceptualization. **Sebastian Fuller:** Writing – review & editing, Writing – original draft, Validation, Supervision, Formal analysis. **Gloria Ngaiza:** Writing – review & editing, Methodology, Formal analysis, Data curation. **Nancy Odinga:** Writing – review & editing, Methodology, Formal analysis, Data curation. **Caroline Waithira:** Writing – review & editing, Methodology, Formal analysis, Data curation. **Vincent Kagonya:** Writing – review & editing, Methodology, Formal analysis, Data curation. **Onesmus Onyango:** Writing – review & editing, Methodology, Formal analysis, Data curation. **Naima Nasir:** Writing – review & editing, Methodology, Formal analysis, Data curation. **Gulraj Grewal:** Writing – review & editing, Methodology, Formal analysis, Data curation. **Abdulazeez Imam:** Writing – review & editing, Methodology, Formal analysis, Data curation. **Asma Rababeh:** Writing – review & editing, Methodology, Formal analysis, Data curation. **Michuki Maina:** Writing – review & editing, Methodology, Formal analysis, Data curation. **Kenneth Karumba:** Writing – review & editing, Methodology, Formal analysis, Data curation. **Maureen Kelley:** Writing – review & editing, Formal analysis, Conceptualization. **Mike English:** Writing – review & editing, Supervision, Methodology, Funding acquisition, Formal analysis, Conceptualization. **Sassy Molyneux:** Writing – review & editing, Writing – original draft, Supervision, Methodology, Investigation, Funding acquisition, Formal analysis, Conceptualization.

## Ethics approval

Ethical approval was given by KEMRI (Kenya Medical Research Institute) Scientific and Ethical Review Committee (SERU) KEMRI/SERU/CGMR-C/229/4203 and the Oxford Tropical Research Ethics Committee (OXTREC) (reference 26-21). We also received approval from the National Commission for Science, Technology and Innovation (NACOSTI/P/23/27504). Additionally, we received approvals from the individual counties and health facilities where the research was conducted. For the HIGH-Q study, informed consent was obtained prior to data collection (observations, interviews and focus group discussions) from all the target participants. To maintain confidentiality, no personal identifiers were collected. We do not include any participant data in this paper.

## Funding

This research was supported by the 10.13039/501100000272NIHR (project references: NIHR130812 and NIHR303168) using UK international development funding from the 10.13039/100013986UK Government to support global health research. The views expressed in this publication are those of the author(s) and not necessarily those of the NIHR or the UK government. 10.13039/100010269Wellcome Trust core awards to the KEMRI-Wellcome Trust Research Programme (227131/Z/23/Z) and (227396/Z/23/Z) and a 10.13039/100010269Wellcome Trust Career Development Award (227329/Z/23/Z) also enabled this research. The funders had no role in study design, data collection and analysis, decision to publish, or preparation of the manuscript.

## Declaration of competing interest

There are no conflicting interests.
